# Rapid and Efficient Generation of Myelinating Human Oligodendrocytes in Organoids

**DOI:** 10.3389/fncel.2021.631548

**Published:** 2021-03-17

**Authors:** Mohammed R. Shaker, Giovanni Pietrogrande, Sally Martin, Ju-Hyun Lee, Woong Sun, Ernst J. Wolvetang

**Affiliations:** ^1^Australian Institute for Bioengineering and Nanotechnology, The University of Queensland, Brisbane, QLD, Australia; ^2^School of Biomedical Sciences, The University of Queensland, Brisbane, QLD, Australia; ^3^Department of Anatomy, Brain Korea 21 Plus Program for Biomedical Science, Korea University College of Medicine, Seoul, South Korea

**Keywords:** oligodendrocyte, myelination, induced pluripotent stem cells, organoid, reporter cell line

## Abstract

Human stem cell derived brain organoids are increasingly gaining attention as an ideal model system for investigating neurological diseases, particularly those that involve myelination defects. However, current protocols for generating brain organoids with sufficiently mature oligodendrocytes that deposit myelin on endogenously produced neurons are lengthy and complicated. Taking advantage of a human pluripotent stem cell line that reports on SOX10 expression, we developed a protocol that involves a 42 day exposure of neuroectoderm-derived organoids to a cocktail of growth factors and small molecules that collectively foster oligodendrocyte specification and survival. Importantly, the resulting day 42 brain organoids contain both myelinating oligodendrocytes, cortical neuronal cells and astrocytes. These oligodendrocyte brain organoids therefore constitute a valuable and tractable platform for functional neurogenomics and drug screening for white matter diseases.

## Introduction

Oligodendrocytes (OL) are cells of the central nervous system (CNS) that generate the multilayered myelin membrane sheath around vertebrate axons, enhancing the propagation of action potentials. OL provide metabolic support to neurons and are important for maintaining axonal integrity (Fünfschilling et al., 2012). In multiple sclerosis, OL generation, turnover and dysfunction have been linked to demyelination, axonal damage and disease progression (Domingues et al., 2016). Similarly, a loss of myelination and a consequent breakdown of OL-axon communication are increasingly linked to neurodegenerative diseases, including amyotrophic lateral sclerosis (Salameh et al., 2015), Parkinson (Ferrer, 2018), and Alzheimer disease (McKenzie et al., 2017). Hypomyelination also occurs in a range of neurodevelopmental diseases such as Down syndrome (Reiche et al., 2019) and is typical of leukodystrophies (Wasseff and Scherer, 2015). Therapies aimed at modulating OL function and survival, as well as delivery of exogenous healthy OL progenitors (OPCs) are promising avenues for restoring axonal integrity and neurological function.

Our current understanding of OL development and function is predominantly based on rodent studies, and to date therapies for treating OL dysfunction have not translated well to human clinical practice (Mak et al., 2014). Recent advances in human brain organoid systems provide an opportunity to study human OL function in health and disease under conditions that more closely mimic the *in vivo* 3D make-up of the brain (Fatehullah et al., 2016). OL fail to be appropriately and efficiently specified in brain organoids subjected to conventional cortical differentiation conditions (Quadrato et al., 2016), and only recently relatively robust induction of oligodendrogenesis and myelination was achieved in organoids, albeit only after a prolonged glial differentiation protocol for 210 days (Madhavan et al., 2018). Since such extended differentiation timelines make experimentation difficult and impractical, Kim et al. pioneered a protocol that accelerates the specification of myelinating OL in organoids and shortened the protocol to 105 days of culture (Kim et al., 2019). Despite this advance rapid high throughput investigation of demyelinating diseases and screening of therapeutics remains cumbersome, even with these reduced timelines. To address this bottleneck, we created a hiPSC line that reports on the expression of the early oligodendroglial gene SOX10 and used this cell line to develop a facile and rapid 42 day protocol for the generation of human brain organoids that contain OL. We show that these OL brain organoids contain OL that myelinate endogenously co-specified cortical neurons and support astrocyte differentiation. Therefore, our efficient and rapid protocol for generating OL brain organoids represents a promising and versatile platform for modeling CNS diseases associated with hypo-myelination or demyelination as well as for drug screening.

## Results

### Generation of Human Brain Organoids With Oligodendrocyte and Neural Progenitors

**Figure 1:**
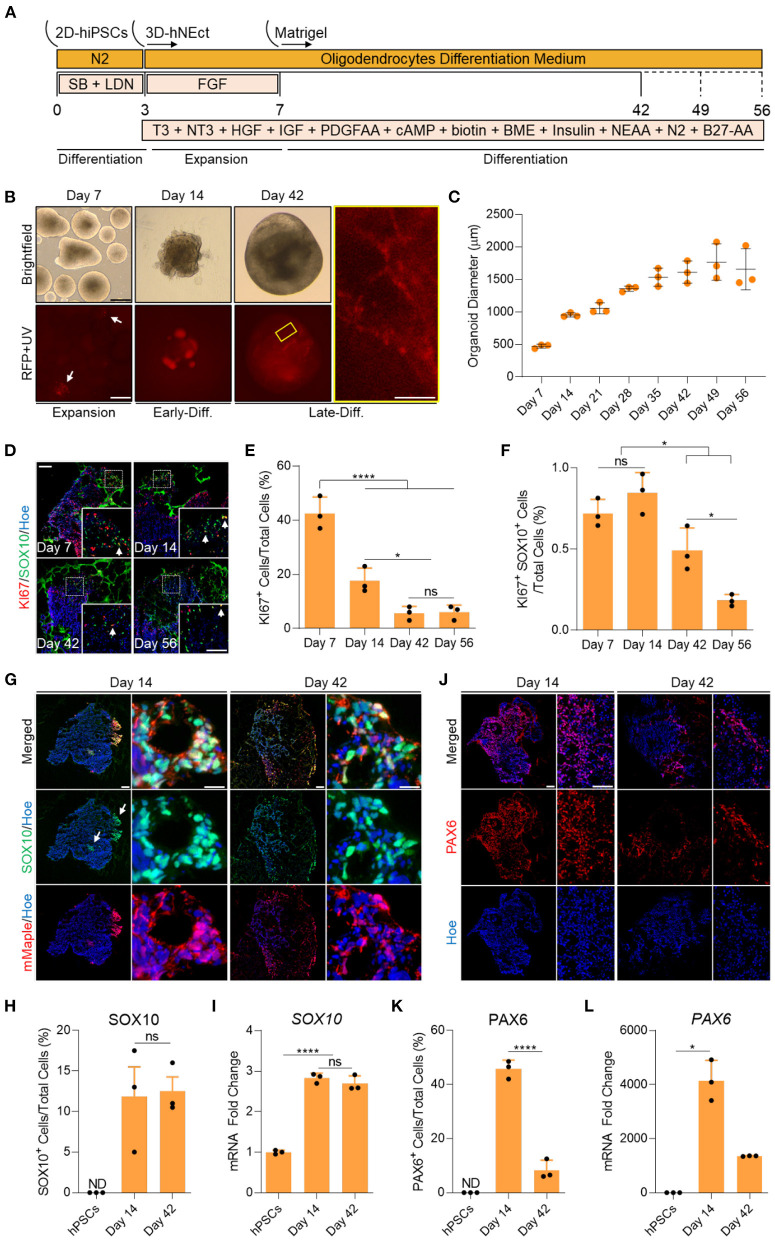
Generation of human brain organoids containing OPCs and NSCs. **(A)** Schematic representation of the strategy used to generate OL brain organoids from hiPSCs. **(B)** Images showing the developmental stages of OL brain organoids over time in culture *in vitro* under brightfield, and red fluorescence protein (RFP). RFP indicates mMaple after UV photoconversion (emission 600–630 nm). Yellow box is a magnified image of a day 42 OL brain organoid. Scale bar is 125 μm, scale bar of magnified image is 15 μm. A total of 24 organoids was analyzed. **(C)** Graph showing the growth of OL brain organoids (based on the average diameter) at different stages of *in vitro* culture. Data are presented as the mean ± standard deviation (*n* = 3). The total number of analyzed organoids is 88. **(D)** Representative images of immunostained sectioned organoids at days 7, 14, 42, and 56 showing protein expression of KI67 (Red) and SOX10 (Green). All sections were counterstained with Hoechst 33342 (Blue). Dotted boxes indicate the magnified images. White arrows indicate cells co-expressing KI67 and SOX10. Scale bar = 200 and 100 μm in magnified images. A total of 48 organoids was analyzed. **(E)** Quantification of the percentage of cells expressing the proliferation marker (KI67) relative to the total number of cells per sample. Data are presented as mean ± standard deviation. Number of independent experiments = 3. *P < 0.05, ****P < 0.001 via One Way ANOVA. NS is not significant. Normality Test (Shapiro-Wilk) Passed (*P* = 0.834). A total of 48 organoids was analyzed. **(F)** Quantification of the percentage of cell co-expressing the proliferation marker (KI67-Red) and OPCs (SOX10-Green) relative to the total number of cells per sample. Data are presented as mean ± standard deviation. Number of independent experiments = 3. *P < 0.05 via One Way ANOVA. NS is not significant. Normality Test (Shapiro-Wilk) Passed (*P* = 0.879). A total of 48 organoids was analyzed. **(G)** Representative images of immunostained day 14 and 42 organoid sections showing the protein expression of SOX10 (Green) and mMaple (Red). All sections were counterstained with Hoechst 33342 (Blue). Scale bar = 200 μm. Scale bar of magnified images 25 μm. A total of 24 organoids was analyzed. **(H)** Quantification of the percentage of total SOX10^+^ cells relative to the total number of cells in organoid sections (six sections, three biological replicates). Data are presented as the mean ± standard deviation. NS indicates the non-significant differences between day 35 and day 56 via One Way ANOVA. ND indicates not detected. Normality Test (Shapiro-Wilk) Passed (*P* = 0.666). A total of 24 organoids was analyzed. **(I)** qRT-PCR of OPC marker (*SOX10*). All values were normalized to GAPDH levels of their respective samples and expressed relative to hiPSC values to obtain the fold change. Data are shown as mean ± standard deviation; Number of independent experiments = 3. Total of 18 organoids were analyzed. ****P < 0.0001 via One Way ANOVA. Normality Test (Shapiro-Wilk) Passed (*P* = 0.542). **(J)** Representative images of immunostained day 14 and 42 organoid sections showing the protein expression of PAX6 (Red). All sections were counterstained with Hoechst 33342 (Blue). Scale bar = 200 μm. scale bar of magnified images 75 μm. A total of 24 organoids was analyzed. **(K)** Quantification of the percentage of total PAX6^+^ cells relative to the total number of cells per sample (six sections, three biological replicates). Data are presented as mean ± standard deviation. Number of independent experiments = 3. ****P < 0.0001 via One Way ANOVA. A total of 24 organoids was analyzed. Normality Test (Shapiro-Wilk) Passed (*P* = 0.808). **(L)** qRT-PCR of NSCs marker (*PAX6*). All values were normalized to GAPDH levels of their respective samples and expressed relative to hiPSC values to obtain the fold change. Data are shown as mean ± standard deviation; Number of independent experiments = 3. Total of 18 organoids were analyzed. *P < 0.05 via Kruskal–Wallis One Way Analysis of Variance on Ranks. Normality Test (Shapiro-Wilk) Failed (*P* < 0.05).

SOX10 expression in the developing CNS is restricted to oligodendroglia (García-León et al., 2018). To track the emergence of oligodendroglia in the developing organoids, we utilized the WTC hiPSCs line (UCSFi001-A) that was engineered to contain an IRES-mMaple fluorescent protein locus (McEvoy et al., 2012) 125 base pairs downstream of the translation stop site in the 3'-UTR of the SOX10 gene, similar to the previously reported Nano-lantern line (Horikiri et al., 2017). To generate brain organoids containing multiple CNS cell lineages, we first generated human neuroectodermal (hNEct) cells by subjecting the hiPSCs for 3 days to a dual SMAD inhibition protocol (Shaker et al., 2020a). These hNEct cells were next lifted off the culture plates and allowed to aggregate in low attachment dishes (Supplementary Video 1) while stimulating their growth with bFGF for 4 days (Figure 1A). Successful formation of the neuroectodermal layer at day 7 was evidenced by bright smooth edges of the spheroids (Figure 1B), and these spheroids were next embedded in Matrigel. From as early as day 3, we exposed the organoids to a cocktail of growth factors and small molecules (Figure 1A), that included thyroid hormone T3, neurotrophin NT3, hepatocytes growth factor (HGF), insulin growth factor (IGF), and platelet derived growth factor (PDGF-AA) to promote differentiation toward the oligodendroglial lineage and to foster the proliferation of newly generated OPCs, as well as B27 without vitamin A and cAMP to drive the maturation of OPCs to myelinating OL (Douvaras and Fossati, 2015; Zhang et al., 2019). We further added biotin and BME to the OL differentiation media to increase the survival of the maturing OL. In addition, we added N2, insulin, and non-essential amino acids to promote the co-differentiation of neurons and astrocytes (Figure 1A).

To identify the proliferation and differentiation phases of OL brain organoid development, we measured the diameter of the organoids over time in culture. OL brain organoids gradually increased in size during the proliferative phase between day 7 and 35. The organoids then entered the differentiation phase where they maintained an average size of 1.5 mm until day 56 in culture (Figure 1C). In agreement with these data, immunostaining for the proliferation marker KI67 revealed 40% and 18% of cells expressed KI67 on day 7 and day 14, respectively, followed by a significant drop to about 5% by day 42 and day 56 (Figures 1D,E). Live imaging of OL brain organoids derived from the SOX10 reporter iPSCs subjected to our differentiation protocol revealed that SOX10 expression can be detected as early as day 7, as indicated by the presence of cells expressing photo-convertible mMaple (Figure 1B, RFP + UV in red, white arrows). Interestingly, we found <1% of SOX10+ cells expressed KI67 at days 7 and 14 which significantly dropped to <0.5% at days 42 and 56 (Figure 1F), consistent with recent single-cell transcriptomic analyses of human OL that revealed a cluster of OPCs are in s-phase and enriched for genes associated with cell cycle and division (Chamling et al., 2021).

The abundance of mMaple expressing cells substantially increased by day 14 and immunofluorescent labeling of organoid sections confirmed that mMaple expressing cells were labeled with SOX10 antibodies (Figure 1G, white arrows). We detected clusters of mMaple/SOX10 expressing cells at the periphery of the organoids by day 14, that subsequently dispersed over the periphery of the organoids by day 42 (Figure 1G). At this timepoint the mMaple labeled cells displayed a predominantly bipolar appearance with long processes, suggestive of OL maturation (Figure 1B, yellow box). This cell distribution pattern did not change with further maturation in culture (Supplementary Figure 1A).

To assess the abundance of oligodendroglia in the organoids, we quantified SOX10 expressing cells at day 14 and found ~11% of OL brain organoid cells expressed SOX10 and that this did not significantly increase by day 42 (Figures 1G,H). In agreement with these data, we detected robust upregulation of *SOX10* mRNA by day 14 and no significant increase by day 42 (Figure 1I). We next assessed whether our optimized media cocktail would allow the co-differentiation of neural stem cells (NSCs) from hNEct cells. Our data show that 45% of cells in the OL brain organoids expressed the NSCs marker PAX6 at day 14 (Figures 1J,K), and that this number dropped to 8% in day 42 OL brain organoids (Figure 1K), in agreement with the notion that these organoids had entered the differentiation phase. A similar pattern was observed for *PAX6* mRNA levels (Figure 1L). Importantly, OL brain organoids exhibited a significant upregulation of the forebrain marker genes *EMX2* and *OTX1* between day 14 and day 42 (Supplementary Figure 1B), indicative of forebrain cortical neuron differentiation in the OL brain organoids. Collectively these data led us to conclude that our OL brain organoid differentiation protocol promotes the rapid specification of OPCs as well as NSCs with the potential to generate forebrain OL and cortical neurons, respectively.

### Progressive Maturation of Oligodendrocytes in OL Brain Organoids

To assess when and to what extent SOX10 expressing cells differentiate into pre-OL, immature OL, and mature myelinating OL, we first investigated the mRNA expression of several genes that mark these different stages of oligodendrogenesis. We found that *O4* expression, which marks pre- and immature-OL, was significantly increased by day 21 of differentiation (Supplementary Figure 2). By day 42, OL brain organoids showed significantly increased expression of *GALC* and *MBP*, genes that are expressed in mature and myelinating OL, respectively (Supplementary Figure 2). We also examined the expression of *OLIG2, NG2* and *NKX2.1* which are known to be expressed in OPCs and regulate oligodendrogenesis (Kessaris et al., 2006; Rivers et al., 2008). Interestingly, we found a significant induction of *OLIG2* and *NG2* at day 42 of differentiation (Supplementary Figure 2), while *NKX2.1*, a transcription factor known to regulate OL lineage specification, was significantly expressed at day 21 and persisted until day 42 of differentiation (Supplementary Figure 2).

**Figure 2:**
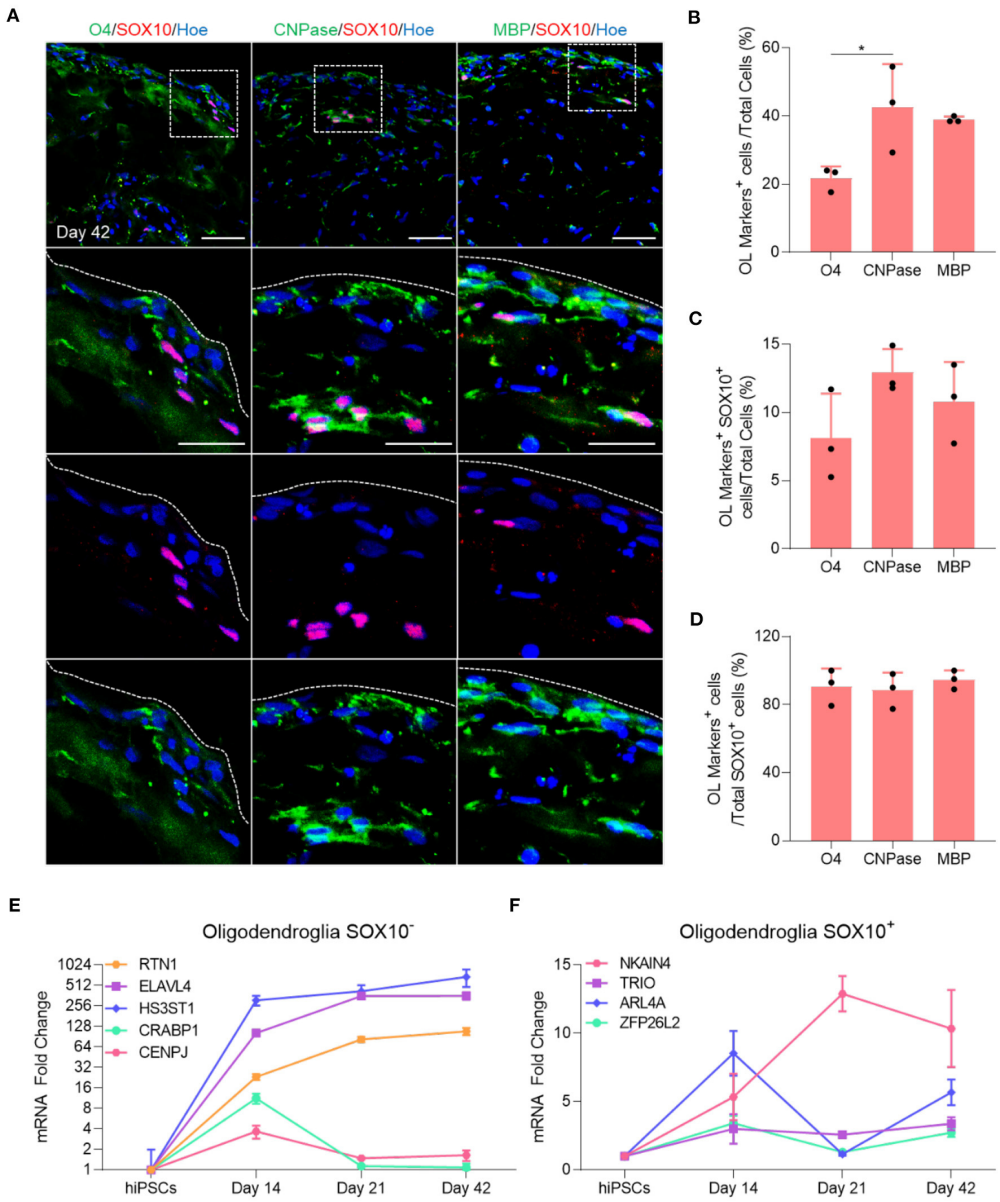
Maturation of oligodendrocytes in brain organoids. **(A)** Representative images of immunostained day 42 organoid sections showing the protein expression of SOX10 (Red) and the oligodendrocytes markers of different stages of maturation labeled with O4 (Green), CNPase (Green), and MBP (Green). All sections were counterstained with Hoechst 33342 (Blue). Dotted boxes indicate the magnified area. Dotted lines indicate the peripheral boundary of the organoid. Scale bar = 50 μm. A total of 48 organoids was analyzed. **(B)** Quantification of the percentage of total oligodendrocyte cells labeled with O4, CNPase and MBP relative to the total number of cells in organoid sections (seven sections, three biological replicates). Data are presented as the mean ± standard deviation. Number of independent experiments = 3. *P < 0.05 via One Way ANOVA. A total of 48 organoids was analyzed. Normality Test (Shapiro-Wilk) Passed (*P* = 0.179). **(C)** Quantification of the percentage of total SOX10 positive oligodendrocyte cells that are labeled with O4, CNPase and MBP relative to the total number of cells in organoid sections (seven sections, three biological replicates). Data are presented as the mean ± standard deviation. Number of independent experiments = 3. A total of 48 organoids was analyzed. Normality Test (Shapiro-Wilk) Passed (*P* = 0.584). **(D)** Quantification of the percentage of total SOX10^+^ oligodendrocyte cells labeled with O4, CNPase and MBP relative to the total number of SOX10^+^ cells in organoid sections (seven sections, three biological replicates). Data are presented as the mean ± standard deviation. Number of independent experiments = 3. Normality Test (Shapiro-Wilk) Passed (*P* = 0.372). **(E)** qRT-PCR of several markers expressed in SOX10 negative oligodendroglial cells. All values were normalized to GAPDH levels of their respective samples and expressed relative to hiPSC values to obtain the fold change. Data are shown as mean ± standard deviation; Number of independent experiments = 3. Total of 18 organoids were analyzed. Y axis is adjusted to log 2 value. **(F)** qRT-PCR of several markers expressed in SOX10 positive oligodendroglial cells. All values were normalized to GAPDH levels of their respective samples and expressed relative to hiPSC values to obtain the fold change. Data are shown as mean ± standard deviation; Number of independent experiments = 3. Total of 18 organoids were analyzed.

Immunofluorescent staining of organoid sections confirmed the expression of O4, CNPase and MBP protein in these organoids (Figure 2A), and automated quantification of these markers in day 42 OL brain organoids revealed that 20% of total cells expressed O4 (Figure 2B), and that 40.7% and 39.4% of all cells were stained with CNPase and MBP antibodies, respectively (Figure 2B). Among these, only 8% of O4-labeled cells, 12% of CNPase-labeled cells and 10% of MBP-labeled cells expressed SOX10 (Figure 2C). Around 90% of all SOX10^+^ cells co-expressed O4, CNPase or MBP, indicating that the vast majority of SOX10 expressing cells were differentiated into the oligodendrocyte lineage (Figure 2D). We speculate that the small number of SOX10 expressing cells that do not express these markers are either immature OPC progenitors or cells of the neural crest lineage that are co-specified.

Multiple single-cell transcriptomic studies of human OL revealed that not all oligodendroglial cells express SOX10 (Chamling et al., 2021), and that other neural cells can act as an additional source of human cortical OPCs (Huang et al., 2020), in agreement with the observation that not all OL generated in our OL brain organoids express SOX10 (Figure 2C). To further examine this possibility, we quantified the expression of several genes that are expressed in SOX10 negative OPCs progeny as well as genes expressed in the SOX10 positive OPCs population, as identified by Chamling et al. (2021). Interestingly, our qPCR analysis revealed that *RTN1, ELAVL4*, and *HS3ST1*, genes expressed in the SOX10 negative OPCs population, commenced at day 14 and persisted throughout subsequent maturation steps, whereas *CRABP1* and *CENPJ* were only expressed at day 14 and subsequently downregulated when mature OL are specified (Figure 2E). Furthermore, we also validated the presence of oligodendroglial genes associated with SOX10 positive OPCs (Figure 2F). Collectively, these data first indicate that the vast majority of OPCs successfully differentiated into mature OL within 42 days of OL brain organoid differentiation and that OL brain organoids contain both SOX10 positive and SOX10 negative OPCs that both contribute to the generation of OL.

### OL Brain Organoids Contain Astrocytes and Oligodendrocytes That Myelinate Endogenously Produced Neurons

**Figure 3:**
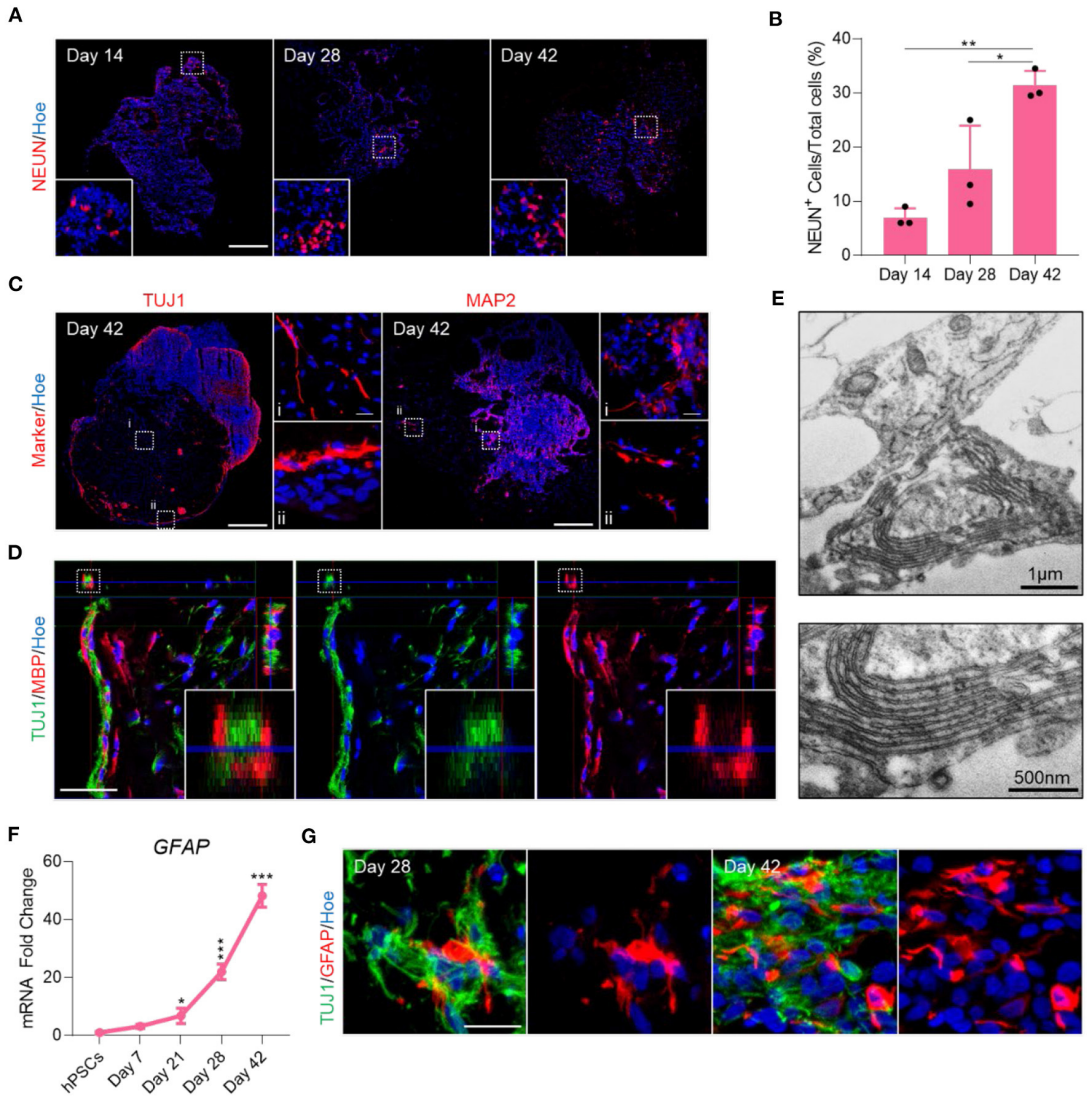
Endogenous generation of myelinated neurons and astrocytes in OL brain organoids. **(A)** Representative images of immunostained day 14, 28, and 42 organoid sections showing the protein expression of NEUN (Red). All sections were counterstained with Hoechst 33342 (Blue). Scale bar = 200 μm. A total of 50 organoids was analyzed. **(B)** Quantification of the percentage of total NEUN^+^ cells relative to the total number of cells in organoid sections (six sections, three biological replicates). Data are presented as the mean ± standard deviation. Number of independent experiments = 3. *P < 0.05. **P < 0.01 via One Way ANOVA. A total of 45 organoids was analyzed. Normality Test (Shapiro-Wilk) Passed (*P* = 0.372). **(C)** Representative images of immunostained day 42 organoid sections showing the protein expression of TUJ1 and MAP2 (Red). All sections were counterstained with Hoechst 33342 (Blue). Dotted boxes indicate the magnified images. Scale bar = 200 μm, magnified scale bar = 17 μm. A total of 50 organoids was analyzed. **(D)** Orthogonally cut images of immunostained day 42 organoid sections showing expression of TUJ1 (Green) and MBP (Red) protein. Dotted boxes indicate the magnified images. All sections were counterstained with Hoechst 33342 (Blue). Dotted boxes indicate the magnified images. Scale bar = 40 μm. **(E)** Transmission electron microscopy of OL brain organoids at day 42 showing evidence of myelin sheath layer formation by the oligodendrocytes. Sections were performed at the edge of organoids where previous immunostaining indicates the interaction between neurons and oligodendrocytes. Scale bar = 1 μm, scale bar of magnified images = 500 nm. **(F)** qRT-PCR of astrocyte marker (GFAP). All values were normalized to GAPDH levels of their respective samples and expressed relative to hiPSC values to obtain the fold change. Data are shown as mean ± standard deviation; Number of independent experiments = 3. Total of 18 organoids were analyzed. ***P < 0.001, *P < 0.05 via One Way ANOVA. Normality Test (Shapiro-Wilk) Passed (*P* = 0.646). **(G)** Representative images of immunostained day 28 and 42 organoid sections showing the protein expression of TUJ1 (Green) and GFAP (Red). All sections were counterstained with Hoechst 33342 (Blue). Scale bar = 20 μm. A total of 48 organoids was analyzed.

CNS myelination *in vivo* requires mature oligodendrocytes, astrocytes and sufficiently mature neurons (Simons and Nave, 2015). To assess whether the OL brain organoids generated with our protocol would be able to recapitulate this process *in vitro*, we first assessed the specification and maturation of cortical neurons. Automated quantification of the mature post-mitotic neuronal marker NEUN in sections from OL brain organoids cultured for increasing amounts of time (Figure 3A) revealed a progressive increase in the number of NEUN positive cells relative to the total cell number from 8% at day 14, 16% at day 28 to 29% at 42 day (Figure 3B). Immunostaining with TUJ1 and MAP2 antibodies revealed labeling of axonal projections and dendrites of cells that were mainly localized to the periphery of OL brain organoid where the majority of mature OL were also localized (Figure 3C). We next assessed whether mature OL would interact with these neurons and engage in myelin deposition. Immunostaining of sectioned OL brain organoids with MBP and TUJ1 revealed that MBP is detected in close proximity to axons, and orthogonally cut sections further demonstrated the sheath-like morphology of MBP encompassing axons, indicative of myelination (Figure 3D). To further verify the presence of myelination in these organoids, we next performed transmission electron microscopy on OL brain organoid sections at day 42 and found a successful formation of compacting myelin sheaths that encircled neuronal axons (Figure 3E). Unlike spinal cord or optic nerve, the axons within the organoids are not aligned and sectioning invariable resulted in oblique cross-sections of the myelinated axons (Supplementary Figure 3A). Quantification of the number of myelin lamellae per axon over 10 randomly chosen axons of OL brain organoid at day 42 revealed a broad variation in myelin lamellae between axons (Supplementary Figure 3B), suggesting progressive events of early myelination in OL brain organoids.

Finally, we assessed the specification of astrocytes in the organoids cultured for progressive periods of time by immunostaining for glial fibrillary acidic protein (GFAP) and via qPCR analysis, revealing upregulation of GFAP mRNA from day 28 onwards (Figure 3F) and the emergence of immature GFAP positive cells at day 28. The number of GFAP expressing cells increased by day 42 and these cells displayed a more mature appearance (Figure 3G). We conclude that OL brain organoids contain OL capable of myelinating endogenously produced cortical neurons and contain progressively maturing astrocytes.

## Discussion

Myelin is produced by OL and is essential for enabling saltatory nerve conduction as well as normal brain function, and myelin disturbance is therefore often associated with neuropathologies and neurodevelopmental disorders. For instance, loss of myelin is a pivotal event in autoimmune disorders such as multiple sclerosis, whereas a failure in myelin formation occurs in leukodystrophies, such as in Pelizaeus-Merzbacher disease (Garbern et al., 1999). OL defects in specific leukodystrophies may be due to defects in OL differentiation, in altered MBP production, or impairment of the mechanism for myelin deposition (Nevin et al., 2017). An increasing body of literature suggests that effective myelination or inappropriate demyelination defects are not only due to defects in oligodendrocytes but can also be caused by suboptimal function of other brain cell types. For example, astrocytes are required for supporting myelination, microglia can trigger demyelination, and neurons themselves need to present appropriate signaling molecules and mechanical cues to trigger axon myelination (Matejuk and Ransohoff, 2020). It is not unlikely that the selective loss or deposition of myelin in specific brain regions, such as occurs in Hypomyelination with Brain Stem and Spinal Cord Involvement and Leg Spasticity (HBSL) (Taft et al., 2013; Fröhlich et al., 2018), is driven by a dysfunction of multiple brain cell types. For these reasons brain organoids in which these critical cooperative cell types are appropriately produced are valuable tools for studying (de)-myelination in health and disease states and constitute potential drug testing platforms for diseases that affect myelination, such as Pelizaeus-Merzbacher disease (Elitt et al., 2018; Nobuta et al., 2019) and HBLS.

To monitor the specification of putative oligodendroglia in brain organoids in real time we engineered the WTC human iPSC cell line to express the photo-convertible fluorophore mMaple under the control of the endogenous SOX10 regulatory system through knock-in into the 3'-UTR, since SOX10 is a pivotal transcription factor that drives oligodendroglial development. Using this cell line, we tested and optimized the composition of a single medium cocktail that is capable of inducing the specification of SOX10 positive OPCs in only 4 days following hNEct differentiation. Importantly, we show that this medium composition allows concurrent cortical development, astrocytes differentiation and neuronal maturation. Our data show that SOX10 expressing OPCs in the OL brain organoids predominantly differentiate into cells of the oligodendrocyte lineage, as indicated by the co-expression of MBP, CNPase and O4 in these cells, and that these cells associate with and deposit myelin on endogenous neurons, as indicated by co-localization of MBP with the neuronal marker TUJ1. In our protocol, the exposure of hNEct to a cocktail of growth factor allowed the emergence of mMaple/SOX10 clusters of cells which later dispersed around the OL brain organoids. Immunostaining of sectioned organoids revealed the peripheral distribution of oligodendroglial cells. We cannot exclude that this peripheral location is, at least in part, attributable to a lack of nutrition and oxygen penetration into the core of the organoids (Mansour et al., 2018), as human OL are known to be particularly sensitive to hypoxic conditions (Gautier et al., 2015).

We noted that there are a substantial fraction of cells that do not express SOX10 yet are labeled with CNPase and MBP antibodies. Since these proteins are also present in myelin sheaths it is likely that these represent neuronal cells that have started to undergo myelination (as shown in Figure 3). Intriguingly, we also detected a proportion of cells that were O4 positive yet SOX10 negative, suggesting that in our human organoids not all oligodendroglial progenitor-derived cells express SOX10. During oligodendrogenesis, oligodendroglial cells progress through multiple stages before specification into mature OL that produce myelin proteins and myelinate neurons. Although many markers have been identified that mark oligodendroglia at different stages of differentiation, there are no unique markers that uniquely distinguish these different stages of oligodendrogenesis (Goldman and Kuypers, 2015). For instance, oligodendroglia at pre-OL stage express SOX10, PLP, O4, O1 and CNPase. These proteins persist until the mature stage when OL begin to express additional markers such as GalC, MBP, MAG, and MOG (Kuhn et al., 2019). In this study, we found that in day 42 OL brain organoids ~90% of SOX10 positive cells express O4, as well as CNPase and MBP. These data suggest that oligodendroglial cells and OL produced in our protocol mimic the *in vivo* events of oligodendrogenesis. It is not unlikely that the remaining 10% of SOX10 positive cells may require additional time *in vitro* to differentiate into mature OL. In our hands, the OL brain organoid differentiation protocol described in this paper is robust as we find similar consistent specification of myelinating oligodendrocytes between different cell lines as well as between different clones of the same line (data not shown).

Oligodendroglial cells mature to deposit myelin around neurons that progressively develop electrophysiological as organoids mature over time. It will be interesting to find out whether the accelerated deposition of myelin as reported here results in faster neuronal maturation or more mature electrophysiological properties of the neurons, and whether this is different in OL organoids established from iPSC derived from patients with hypomyelination disorders. While we observe clear evidence of myelin sheaths wrapping around axons, we acknowledge that in day 42 and day 56 organoids we detect only very few axons with compacted myelin sheaths that are observed *in vivo*, similar to previous reports (Madhavan et al., 2018; Kim et al., 2019). It remains to be established whether more prolonged culture or addition of myelination enhancing molecules such as lanosterole, ketoconazole, and clemastine can further enhance neuronal myelination and the development of more compact myelin sheaths in our OL brain organoids.

Previously the pioneering work by Madhavan et al. (2018) demonstrated that generating brain organoids with myelinating oligodendrocytes and elucidation of myelination associated disease processes is indeed possible, however, the organoid differentiation process required 210 days and timed addition of various growth factor cocktails. Kim et al. (2019) subsequently showed that this could be accelerated and observed MBP expressing mature OL as early as 9 weeks and myelination of axons by 15 weeks. In this paper, we now describe an innovative and simplified one step protocol for generating human cortical brain organoids that contain neurons and astrocytes as well as oligodendrocytes that are able to myelinate endogenous neuronal axons by 42 days, making this protocol to our knowledge the fastest method to date for generating mature oligodendrocytes within a human cortical organoid.

## Materials and Methods

### Engineering Human Induced Pluripotent Stem Cells

For gene targeting WTC cells at passage 22 were dissociated in 1 mL Accutase, collected into a 15-mL Falcon, centrifuged (160 × g, 3 min, RT) and counted manually. Three million cells were resuspended in 100 μL of Lonza Amaxa Primary P3 nucleofection solution with 3 μg vector DNA for mMaple transgene insertion and 1 μg of a plasmid expressing the guide RNA and CRISPR-Cas9 (pSpCas9(BB)-2A-Puro (PX459) V2.0 was a gift from Feng Zhang, Addgene plasmid # 62988) and subjected to nucleofection following the manufacturer's instructions for the Lonza Amaxa Primary P3 Kit (V4XP-3024, Lonza). After nucleofection cells were replated onto a 6 well plate in mTser Plus supplemented with 10 μM ROCKi Y-27632 (Lonza-PeproTech, 1293823-B) for 24 h. After 72 h cells were incubated with mTeSR Plus containing 0.5 ug/ml of Puromycin (Life Technologies, A1113803) for 72 h. After further 5 days of culture cells were detached with Accutase and single cell cloned. After 2 weeks of expansion individual clones were screened and frozen in Synth-a-Freeze (Thermofisher, A1254201).

### Human iPSCs Culture and OL Brain Organoid Generation

Engineered mMaple WTC line was cultured according to Stem Cell Technologies protocols (https://www.stemcell.com/maintenance-of-human-pluripotent-stem-cells-in-mtesr1.html) on Matrigel (StemCell Technologies, Cat. #354277) in mTeSR (Stem Cell Technologies, Cat. #85851) (Shaker et al., 2020a). To generate OL organoids, iPSCs were first treated with 10 uM SB 431542 (Sapphire Biosciences, A10826) and 0.1 uM LDN193189 Dihydrochloride (Sigma, SML0559) for 3 days in N2 medium: DMEM/F12 (Gibco, Cat. #11320-33), 2% B-27 supplement (Gibco, Cat. # 17504044), 1% N-2 supplement (Gibco, Cat. #17502-048), 1% MEM Non-Essential Amino Acids (Gibco, Cat. #11140-050), 1% penicillin/streptomycin (Gibco, Cat. #15140148), 0.1% β-mercaptoethanol (Gibco, Cat. #21985-023) to induce hNEct cells (Shaker et al., 2020c). These cells were then detached gently using dispase enzyme for 2o min at 37°C and allowed to aggregate in low-attachment culture plates (Sigma, CLS3473) overnight (Lee et al., 2020b), and expanded for 4 days in OL differentiation medium (OLDM) consisting of: DMEM/F12 (Gibco, Cat. #11320-33), 2% B-27 minus vitamin A supplement (Gibco, Cat. # 17504044), 1% N-2 supplement (Gibco, Cat. #17502-048), 1% MEM Non-Essential Amino Acids (Gibco, Cat. #11140-050), 1% penicillin/streptomycin (Gibco, Cat. #15140148), 0.1% β-mercaptoethanol (Gibco, Cat. #21985-023), Human IGF-I (Lonza-PeproTech, 100-11-100), Insulin (LifeTechnologies, 12585014), Human NT-3 (Peprotech, 450-03-50), 3,3',5-Triiodo-L-thyronine (Sapphire Bioscience, 000-23845), HGF (Lonza-PeproTech, 100-39H) Biotin (Sigma, B4639) cAMP (Sigma, D0627), PDGF-AA (Lonza-PeproTech, 100-13A). Induced neuroepithelial spheroids were then embedded in Matrigel (StemCell Technologies, Cat. #354277) and maintained in OLDM medium. Fresh media was replaced three times a week. To verify the induction of SOX10 in OL organoids, organoids were exposed to UV light for 30 s for efficient mMaple photoconversion. Brightfield images were automatically acquired with 582 JuLi™Stage (NanoEntek) to capture stages of hNEct 2D sheet conversion into 3D. All experiments were carried out in accordance with the ethical guidelines of the University of Queensland and with the approval by the University of Queensland Human Research Ethics Committee (Approval number-2019000159).

### Immunohistochemistry

Tissue processing was performed as described in Lee et al. (2019) and immunohistochemistry (IHC) was performed as described in Kim et al. (2017). In brief, organoids were fixed in 4% PFA for 1 to 3 h at room temperature (RT) or overnight at 4°C. Fixed organoids were then washed three times for 10 min at RT with 1 × phosphate buffered saline (PBS) before immersing in 30% sucrose in 1 × PBS at 4 °C. Organoids were allowed to sink before embedding in a solution containing 3:2 ratio of Optimal Cutting Temperature (O.C.T) and 30% sucrose on dry ice. Mounted blocks of tissues were then sectioned into 14-μm thick serial sections, and collected onto Superfrost slides (Thermo Scientific, cat. #SF41296) (Shaker et al., 2020b). For IHC (Lee et al., 2020a), organoid sections were washed with 1 × PBS three time for 10 min at RT before blocking for 1 to 6 h at RT with 3% bovine serum albumin (BSA) (Sigma, Cat. A9418-50G) and 0.1% triton X-100 in 1x PBS. After blocking, the sectioned organoids were incubated with primary antibodies overnight at 4°C, followed by washing with 1 × PBS three times for 10 min each at RT. Labeled tissues were then incubated with appropriate secondary antibodies for 1 h at RT before mounting and imaging. All samples were counterstained with Hoechst 33342 (Invitrogen, Cat. #H3570). All images were acquired using confocal microscopy (Leica TCS SP8). The primary antibodies used in this study are listed in Supplementary Table 1. Alexa-488, Alexa-546, and Alexa-633-conjugated secondary antibodies were obtained from Jackson ImmunoResearch Laboratory.

### qRT-PCR

Total RNA was isolated from 3 pooled organoids using an RNA extraction kit (Qiagen, 79256) according to the manufacturer's instructions (Shaker et al., 2015). cDNA was synthesized starting from 500 ng of RNA using VILO cDNA Synthesis Kit. qPCR was performed using PowerUp SYBR Green Master Mix (Applied Biosystems) on a Bio-Rad CFX96 Touch Real-Time PCR detection system. Each reaction was performed in triplicate. GAPDH was used for normalization. Primers sequences are listed in Supplementary Table 2.

### Transmission Electron Microscopy

Organoids were fixed in 2.5% glutaraldehyde for 24 h. Fixed organoids were contrasted with 1% osmium tetroxide and 2% uranyl acetate prior to dehydration through a series of ethanol solutions (50–100%) and embedded in EPON resin using a PELCO BioWave (Ted Pella Inc). Following polymerisation at 60°C for 24 h, ultrathin sections (~60 nm) were cut using an ultramicrotome (UC64, Leica). Sections were visualized on a transmission electron microscope (model 1011; JEOL) equipped with cooled charge-coupled device camera (Morada; Olympus) and images acquired using iTEM software (Olympus Soft Imaging Solutions).

### Statistical Analysis

All data were expressed as the mean ± standard deviation of the mean of the indicated number of independent experiments. The number of biological replicates as well as the sample size are indicated in the figure legends. Three is the minimum number of replicates used in this study. Image J was utilized to quantify the number of positive cells from confocal images. One-way ANOVA was applied for comparing more than two groups for normally distributed data that passed the normality test (Shapiro-Wilk). Kruskal–Wallis H test or one-way ANOVA on ranks was applied for comparing more than two groups for non-normally distributed data that failed to pass the normality test (Shapiro-Wilk). Tukey's *post-hoc* analysis was applied for comparisons to a single control. Statistical analysis was performed using GraphPad Prism 8.3.1® software. Minimal statistical significance was defined at *P* < 0.05.

## Data Availability Statement

The raw data supporting the conclusions of this article will be made available by the authors, without undue reservation.

## Ethics Statement

All experiments were carried out in accordance with the ethical guidelines of the University of Queensland and with the approval by the University of Queensland Human Research Ethics Committee (Approval number-2019000159).

## Author Contributions

MS: performed, analyzed and designed experiments, interpreted the results, and wrote the manuscript. GP: performed additional experiments and wrote the manuscript. SM: performed additional experiments. J-HL: performed additional experiment. WS: resources. EW: conceived and supervised the study, interpreted results, and wrote the manuscript. All authors: final version of the manuscript was approved.

## Conflict of Interest

The authors declare that the research was conducted in the absence of any commercial or financial relationships that could be construed as a potential conflict of interest.

## Supplementary Material

Supplementary Figure 1 Characterization of mMaple+ cells and regional markers in OL brain organoids, related to Figure 1. **(A)** Analysis of sectioned organoids at days 7, 14, 42, and 56 showing mMaple after UV photoconversion (emission 600–630 nm). All sections were counterstained with Hoechst 33342 (Blue). Scale bar = 200 μm. Total of 48 organoids were analyzed. **(B)** qRT-PCR of forebrain markers (*EMX2* and *OTX1*). All values were normalized to GAPDH levels of their respective samples and expressed relative to hiPSCs values to obtain the fold change. Data are shown as mean ± standard deviation; Number of independent experiments = 3. Total of 18 organoids were analyzed. ^*^*P* < 0.05 via Kruskal–Wallis One Way Analysis of Variance on Ranks. Normality Test (Shapiro-Wilk): Failed.

Supplementary Figure 2 Characterization of oligodendrocytes marker expression in OL brain organoids, related to Figure 2. qRT-PCR of different stages of oligodendrocyte specification markers (O4, GALC, MBP, OLIG2, NG2, and NKX2.1). All values were normalized to GAPDH levels of their respective samples and expressed relative to hiPSC values to obtain the fold change. Data are shown as mean ± standard deviation; Number of independent experiments = 3. Total of 18 organoids were analyzed. ^*^*P* < 0.05, ^**^*P* < 0.01, ^****^*P* < 0.0001 via One Way ANOVA, Normality Test (Shapiro-Wilk): Passed (*P* = 0.667). For MBP, Kruskal-Wallis One Way Analysis of Variance on Ranks was applied. Normality Test (Shapiro-Wilk): Failed (*P* < 0.050).

Supplementary Figure 3 Progress of myelination in OL brain organoids, related to Figure 3. **(A)** Transmission electron microscopy analysis demonstrates the presence of multiple myelin sheaths in day 42 OL brain organoids. Scale bar = 500 nm. **(B)** Plot graph demonstrates the variation in number of myelin lamellae around axons across 42 days OL brain organoids.

Supplementary Table 1 List of Antibodies used for immunohistochemistry.

Supplementary Table 2 List of Primer Sequences used for RT-PCR (5′-3′ orientation).

Supplementary Video 1 Live imaging of induced human NEct 2D sheet conversion to 3D. Induced colonies of hNEct were detached from the dish and transferred to ultra-low attachment culture dish. 2D colonies were converted to 3D hNEct spheroids within 12 h. Serial images were captured every 5 min.
