# Supplementary material for: Rapid and Efficient Generation of Myelinating Human Oligodendrocytes in Organoids
Source: Front Cell Neurosci. 2021 Mar 17;15:631548. doi: 10.3389/fncel.2021.631548 (PMC8010307; doi:10.3389/fncel.2021.631548)

**Table S1.** List of Antibodies used for immunohistochemistry

| **Antigen** | **Host Species** | **Source** | **Cat#** | **Dilution** |
| --- | --- | --- | --- | --- |
| SOX10 | Rabbit | Cell Signaling | mAb #89356 | 1:300 |
| SOX10 | Mouse | R&D Systems | MAB2864 | 1:300 |
| PAX6 | Mouse | Santa Cruz | SC-81649 | 1:500 |
| KI67 | Rabbit | Invitrogen | PA1-21520 | 1:300 |
| O4 | Mouse | Merck | MAB345 | 1:300 |
| CNPase | Mouse | Sigma-Aldrich | C5922 | 1:300 |
| MBP | Rabbit | Cell Signaling | mAb #78896 | 1:300 |
| NEUN | Mouse | Milipore | MAB377 | 1:500 |
| TUJ1 | Rabbit | Cell signaling | D71G9 | 1:1000 |
| MAP2 | Rabbit | Invitrogen | PA5-17646 | 1:400 |
| GFAP | Rat | ThermoFisher | 13-0300 | 1:500 |

**Table S2.** List of Primer Sequences used for RT-PCR (5′−3′ orientation).

| **Gene** | **Primer (Forward)** | **Primer (Reverse)** |
| --- | --- | --- |
| *SOX10* | CCTTCATGGTGTGGGCTCAGG | CGCTTGTCACTTTCGTTCAGCAG |
| *PAX6* | GCCCTCACAAACACCTACAG | TCATAACTCCGCCCATTCAC |
| *OLIG2* | TCCACCAAGAAGGACAAGAAG | CTAGCGTTCTGCCCAGATACTT |
| *GALC* | CTCAGTGCCGTCTGTTGTC | TACCCAGAGCCCTATCGTTC |
| *MBP* | GACTATCTCTTCCTCCCAGC | AGACCAGGATTTGGCTACGG |
| *NKX2.1* | AGCACACGACTCCGTTCTC | GCCCACTTTCTTGTAGCTTTCC |
| *NG2* | GCCACGTTGTCAGTCGATG | CCCATAGGGGACCTCTAGGG |
| *EMX2* | CAGAGTCTTCGCCGCATTCC | AAGGCTGGAACACGCCTTTG |
| *OTX1* | CAAGATCAACCTGCCGGAGT | AAGTAGGAAGAGGAGGGCGT |
| *O4* | GTGGCGGATCGAGTTCTTC | CTCATCAGCCAGGCCATTG |
| *NEUN* | CCAAGCGGCTACACGTCTC | CGTCCCATTCAGCTTCTCCC |
| *GFAP* | CTGCGGCTCGATCAACTCA | TCCAGCGACTCAATCTTCCTC |
| *ELAVL4* | AACCTCTATGTTAGCGGCCTT | TGGACACTCCTGTGACTTGAT |
| *RTN1* | GACCTGTTGTATTGGCGGGAC | GTAGATGCGGAAACTGATGGTG |
| *HS3ST1* | TTTCACGTCGCCCAAAGTG | TGGGTGTAGTCAGATAGCACG |
| *CRABP1* | ACGCAAGTGCAGGAGTTTAG | CGGGTCCAGTAGGTTTTGGG |
| *CENPJ* | TCTCGGGCTGGGGTCATATTA | GAAAGGCTGTATGGGTTTCAGA |
| *NKAIN4* | GGACAGCGAGCTACTGACC | GGCACCTCCTCATGCAGAC |
| *TRIO* | AAACAGCTACACAGAGATTGGG | ACACGTTCATACAGTTCATGGC |
| *ARL4A* | TGTTGTGGACTCTGTTGATGTC | AGCTCAGTTCACCCATTGCTA |
| *ZFP36L2* | GAGAACAAATTCCGGGACCG | GCGTGGAGTTGATCTGGGAG |
| *GAPDH* | AGCCACATCGCTCAGACAC | GGAGAGGCTCACAGTACCTG |

**Supplementary Figures**

Figure S1


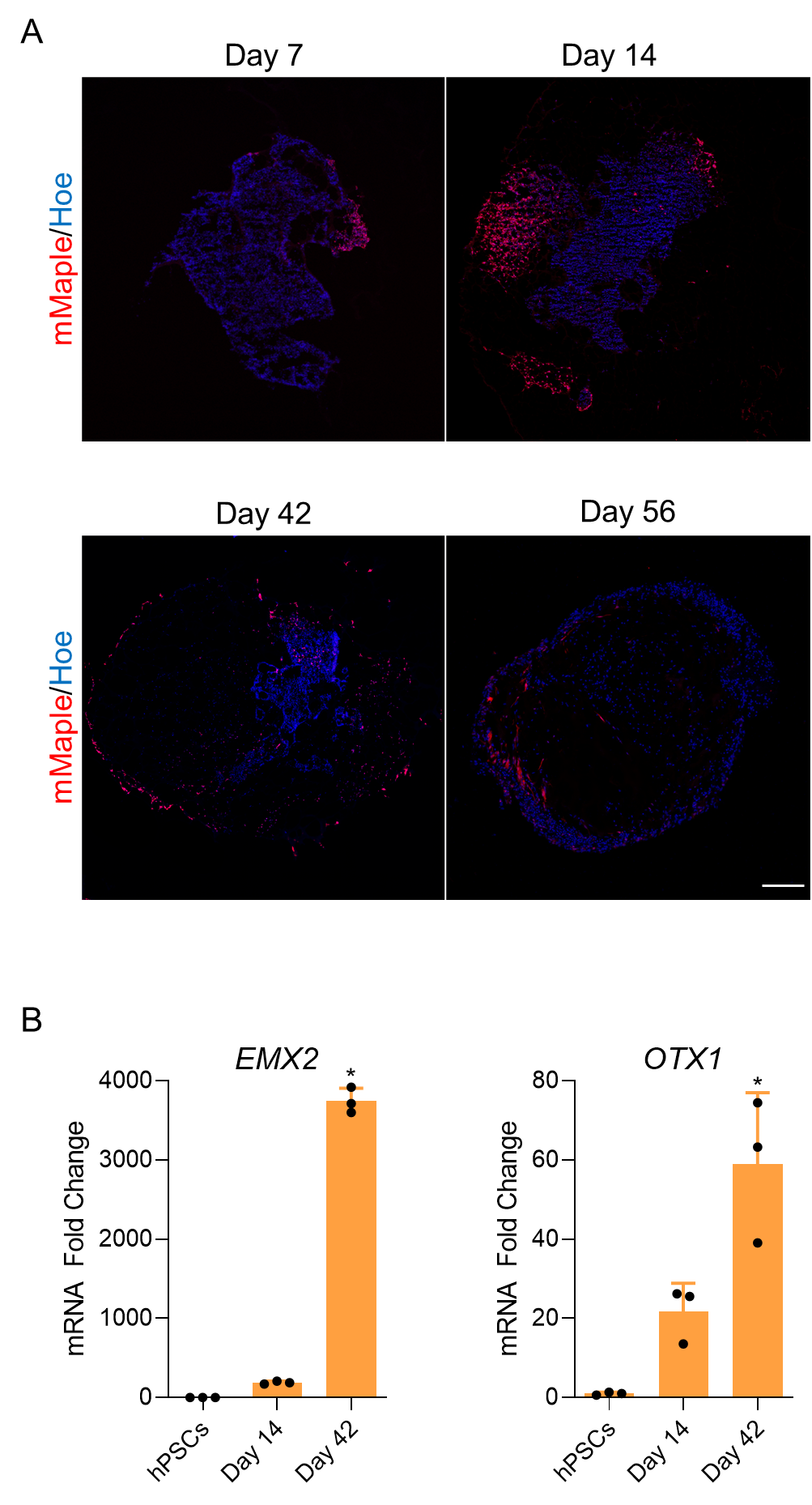


Figure S2


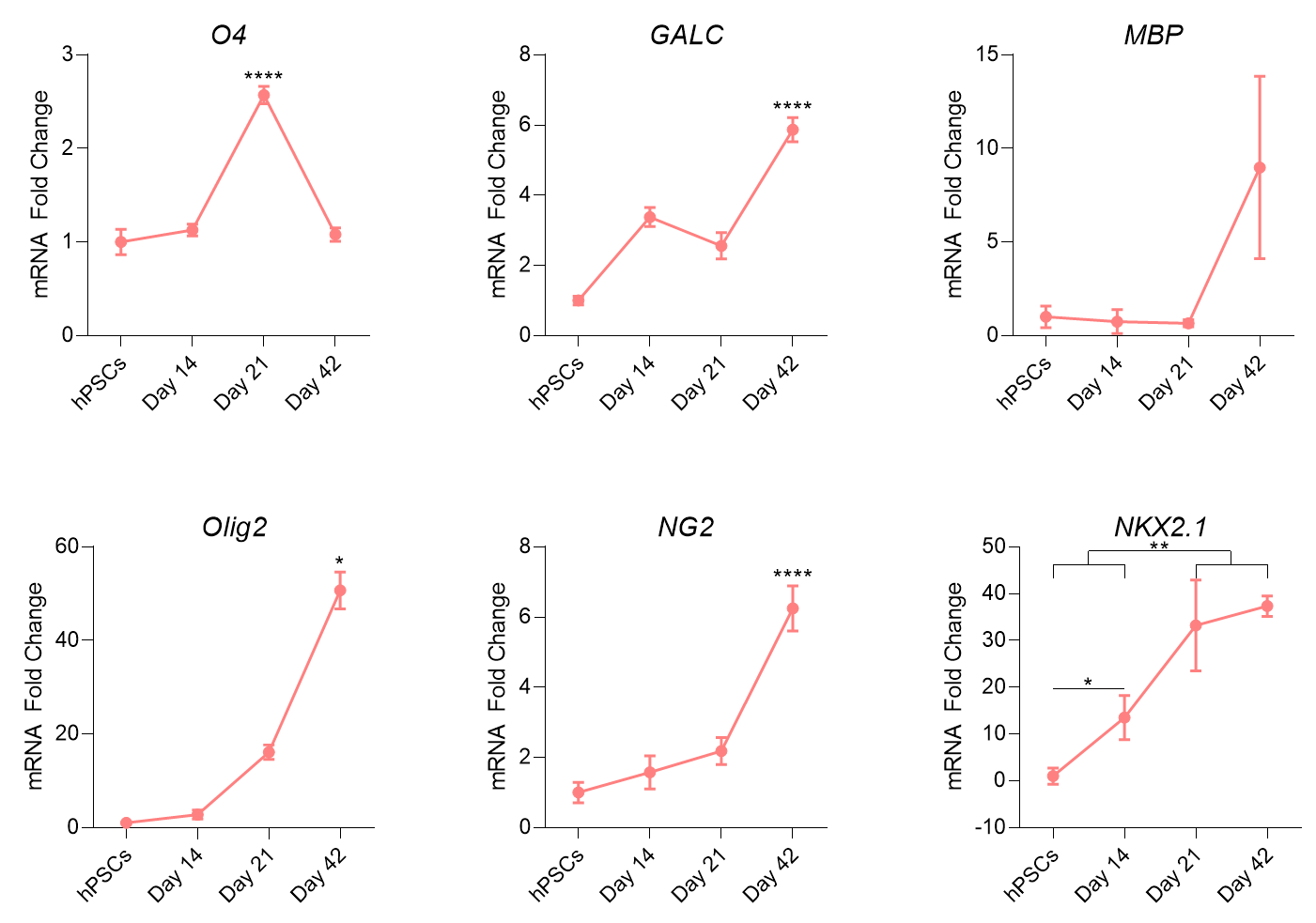


Figure S3


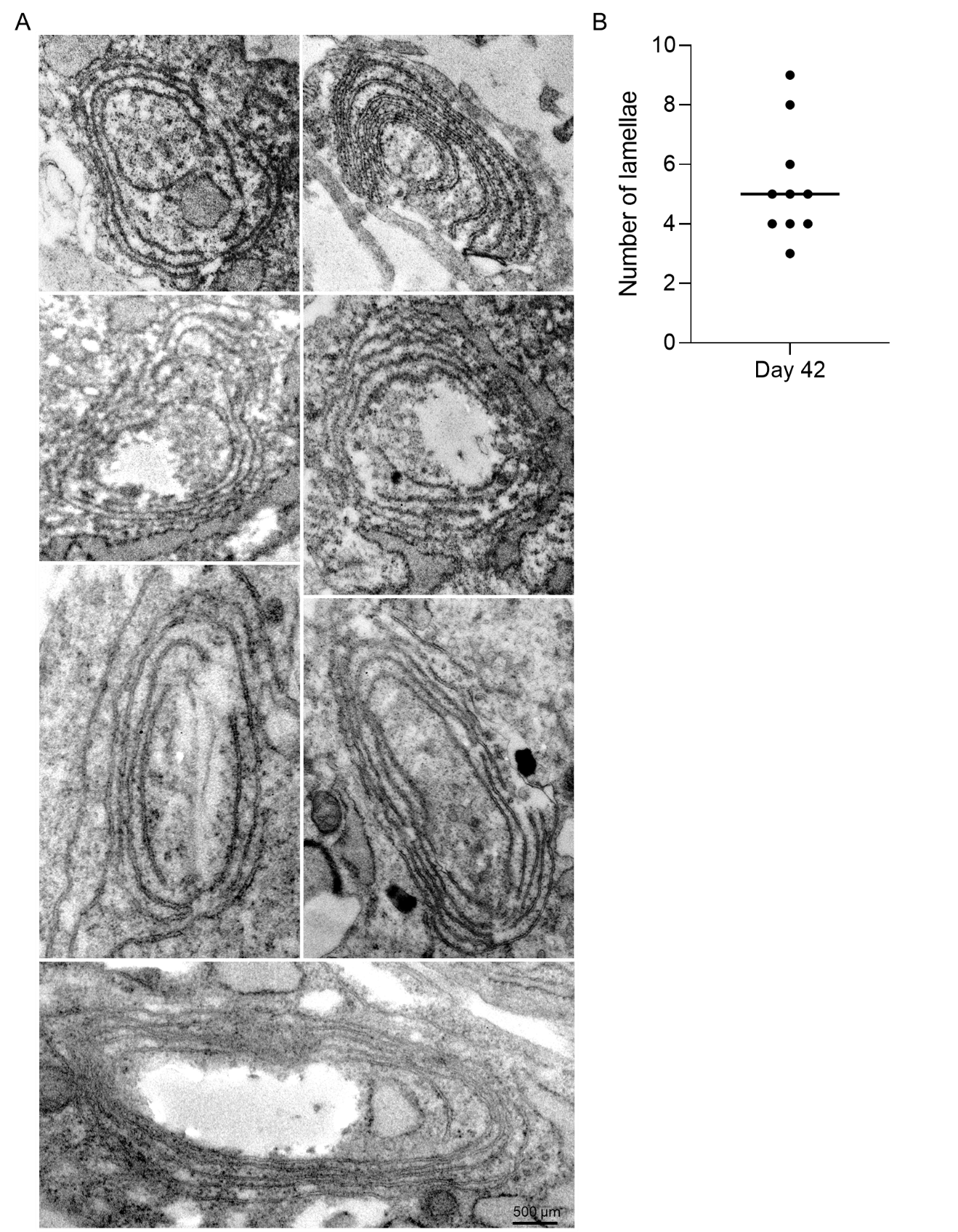

Supplement: Supplementary Figure 1 — Characterization of mMaple+ cells and regional markers in OL brain organoids, related to Figure 1. (A) Analysis of sectioned organoids at days 7, 14, 42, and 56 showing mMaple after UV photoconversion (emission 600–630 nm). All sections were counterstained with Hoechst 33342 (Blue). Scale bar = 200 μm. Total of 48 organoids were analyzed. (B) qRT-PCR of forebrain markers (EMX2 and OTX1). All values were normalized to GAPDH levels of their respective samples and expressed relative to hiPSCs values to obtain the fold change. Data are shown as mean ± standard deviation; Number of independent experiments = 3. Total of 18 organoids were analyzed. *P < 0.05 via Kruskal–Wallis One Way Analysis of Variance on Ranks. Normality Test (Shapiro-Wilk): Failed. [file Data_Sheet_1.docx]
